# Fat, flames and ultrasounds: the effects of obesity on pediatric joint inflammation

**DOI:** 10.1186/s13052-025-01937-5

**Published:** 2025-03-24

**Authors:** Armando Di Ludovico, Ilaria Mascioli, Saverio La Bella, Giovanni Grassi, Concetta Mastromauro, Luciana Breda, Francesco Chiarelli, Anna Maria Musolino, Cosimo Giannini, Antonio Corsello

**Affiliations:** 1 Department of Pediatrics, University of Chieti “G. D’Annunzio”, Chieti, Italy, Via dei Vestini, Chieti, Italy; 2 UOC Rheumatology and Autoinflammatory Diseases, IRCCS Istituto Giannina Gaslini, Genova, Italy; 3 Department of Radiology, Azienda Ospedaliero Universitaria di Cagliari– Polo di Monserrato, Cagliari, Italy; 4 Department of Pediatric Emergency Medicine, Bambino Gesù Children’s Hospital, IRCCS, Rome, Italy; 5 University of Milan, Milan, Italy

**Keywords:** Pediatric obesity, Joint inflammation, Hoffa's fat pad, Ultrasounds, Proinflammatory cytokines, Rheumatology, Metabolic syndrome

## Abstract

The association between childhood obesity and the early appearance of joint degeneration, particularly in the infrapatellar “Hoffa’s” fat pad, highlights the importance of early diagnosis and treatment. The purpose of this review is to describe the role of ultrasound imaging as a first-line imaging tool for the early detection, prevention, and follow-up of degenerative structural changes in children’s joints. By combining ultrasound findings with clinical assessments and indices, healthcare providers can gain a more comprehensive understanding of obesity-related joint alterations. This integrative approach enables early therapeutic interventions, improving outcomes for affected children. Proactive management of pediatric obesity will not only improve the long-term outcomes of obesity-related joint disorders but also reduce the burden of related complications, such as osteoarthritis, in adulthood.

## Introduction

**Fig. 1:**
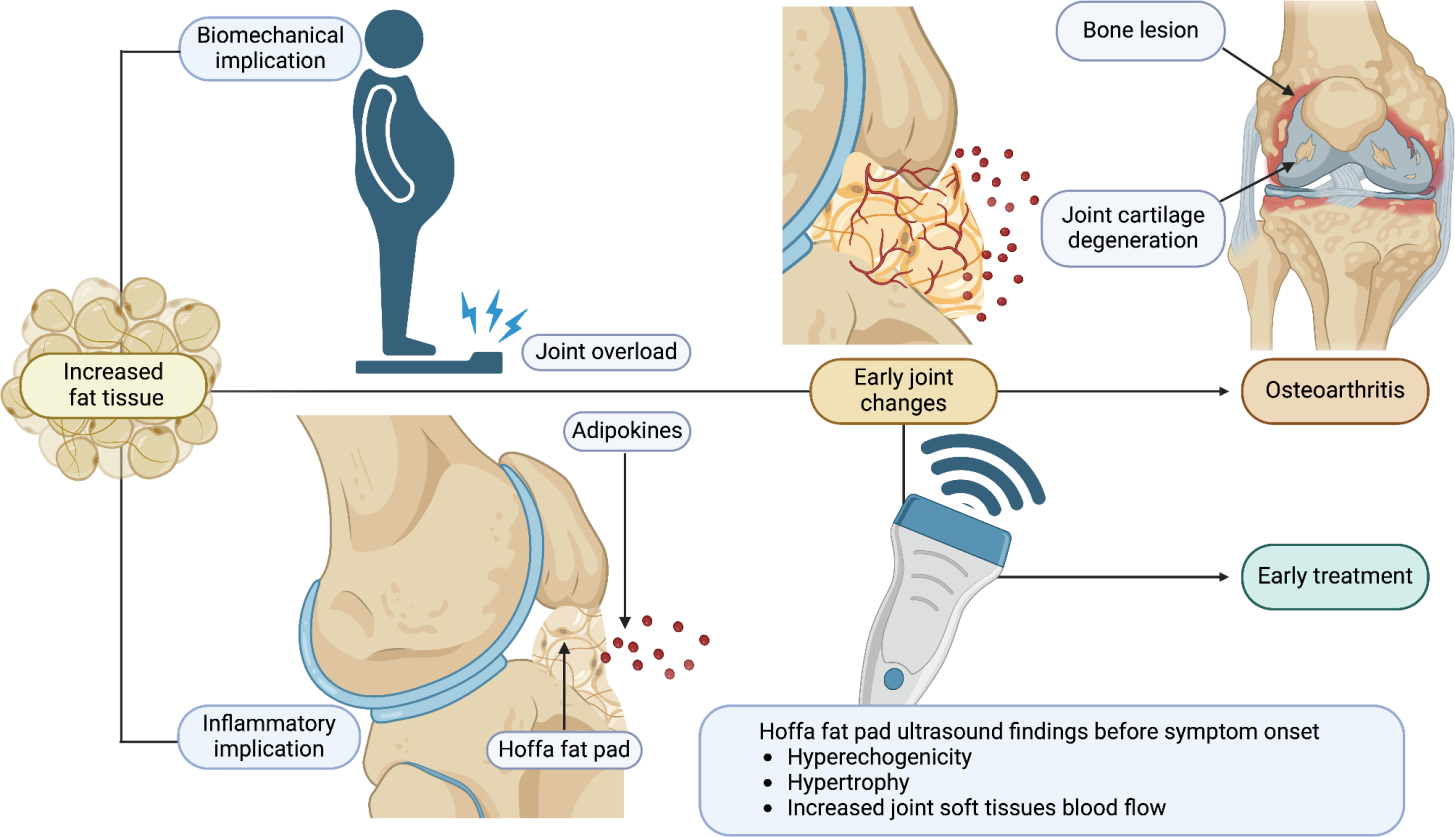
Ultrasound findings may provide a “window” for early treatment of the progression of knee osteoarthritis. Biomechanical effects of increased fat tissue surrounding the joint result in early changes in the joint, which culminate in cartilage breakdown over time

Pediatric obesity has become a relevant concern for public health, with immediate and long-term implications for health outcomes [1]. In addition to its recognized association with metabolic changes, the consequences of childhood obesity on musculoskeletal health and joint integrity are increasingly being diagnosed [2]. In children with obesity, excessive adipose tissue promotes early degenerative changes in load-bearing joints and the pro-inflammatory status, which can further exacerbate these degenerative processes, particularly in the knee [3, 4]. Located immediately posterior to the patellar tendon, the infrapatellar Hoffa’s fat pad is the largest of the anterior knee fat pads and plays an important role in knee biomechanics and stability through shock absorption. Moreover, it is also a metabolically active tissue that secretes inflammatory cytokines under the influence of obesity, leading to joint inflammation and damage [5, 6]. The fat pad of overweight children is prone to chronic inflammation due to the accumulation of cytokines, such as IL-6 and TNF-α, in macrophages [7]. In addition to mechanical stress, these cytokines promote the breakdown of cartilage and inflammation in the synovium, which can lead to joint dysfunction [8]. Ultrasound (US) imaging is a first-line noninvasive technique for assessing Hoffa’s fat pad that provides important findings concerning its structure and inflammatory status [9]. In children with obesity, Hoffa’s fat pad increases in size, and hyperechogenicity is associated with inflammatory markers and signs of joint dysfunction [10]. These imaging findings highlight the need for early identification and treatment of childhood obesity to prevent or reduce joint disorders, also demonstrating the effectiveness of US in this context as a diagnostic and monitoring tool [11]. This review describes successful strategies for preventing and managing joint-related conditions related to childhood obesity, combining clinical approaches and the most recent applications of musculoskeletal US in this field. US is an important tool in the early identification of these alterations and in assessing the effects of therapeutic measures, thereby guiding clinical decisions and management strategies (Fig. 1).

## Impact of childhood obesity on joint health

Childhood obesity is a global epidemic affecting 10% of children worldwide and has rates as high as 20–25% in Mediterranean countries such as Italy and Greece [1]. According to recent data, childhood obesity has nearly tripled in the past four decades and continues to increase [12]. The implications for musculoskeletal health from this alarming trend are profound. Notably, children with obesity have up to a 30% greater risk of knee joint inflammation than their non-obese peers do, and some studies have reported a higher prevalence of severe obesity in adolescents [2]. In these patients, the infrapatellar Hoffa’s fat pad has become a major site of pathological change, playing a role in joint damage through biomechanics and metabolic changes [13]. Longitudinal studies have also shown that obesity in childhood not only predisposes individuals to early musculoskeletal degeneration but also has long-term consequences for joint health, dramatically increasing the risk of developing osteoarthritis in adulthood [14]. Moreover, recent epidemiological studies indicate that joint problems or knee pain affect over 30% of children with obesity, finding a causal relationship between childhood obesity osteoarthritis; however, comprehensive data remain scarce, highlighting a significant gap in current literature [15, 16].

Ultrasound can determine fat localization and integrating it with indices such as the fat mass index (FMI) can be very useful. The FMI is a scale that is calculated in much the same way as BMI; however, it is targeted specifically at fat mass, taking into account variations in body fat that BMI might overlook. It is calculated by dividing an individual’s fat mass, assessed by imaging or bioelectrical impedance analysis, by their height squared [17]. Another scale is the body adiposity index (BAI), which often concerns tools aimed at obesity measurement. The BAI calculates the proportion of body fat in relation to the patient’s hip and height. Even though BAI is an alternative to the widely used BMI, it adds another aspect to body composition, not just weight and height [18]. Nonetheless, these instruments are usually confined to more specialized clinical settings. For general population studies and routine health assessments, BMI and waist circumference are still the gold standards because of their simplicity and well-established associations with health risks [19]. However, US is not the primary tool for evaluating obesity in the general population, but it provides an insightful and detailed view of body fat distribution for clinical assessments, research, and patient care, especially when combined with indices such as the FMI and BAI [20].

## Ultrasound findings

US imaging has revolutionized the diagnosis and follow-up of pediatric musculoskeletal disorders allowing healthcare providers to observe even minor changes in tissue structure and composition [21]. High-frequency transducers, working in the range of 10–20 MHz, make it possible to evaluate Hoffa’s fat pad and the surrounding knee joint with great precision, highlighting even subtle changes [22, 23].

Furthermore, advancements in technology have significantly improved the sensitivity of color-Doppler sonography, enabling the detection of even low-velocity blood flows. This enhancement is particularly valuable for assessing neovascularization and changes in blood flow dynamics in inflamed tissues, which are key features of pediatric musculoskeletal disorders [24]. Elastosonography adds another layer of depth to this method for evaluating tissue rigidity and providing valuable information about joint biomechanics [25]. Integrating diagnostic US imaging with clinical and laboratory data is crucial for developing more effective treatment plans and better outcomes among children with musculoskeletal disorders.

Hoffa’s fat pad is particularly affected by obesity, where chronic mechanical overload and systemic inflammation contribute to pathological changes, including synovitis and tissue degeneration. If untreated, these changes can lead to persistent joint inflammation, mobility limitations, and broader impacts on a child’s overall health and social well-being [4]. US in obesity assessment enables precise measurement of subcutaneous adipose tissue thickness, with a strong correlation (*r* = 0.697–0.907, *p* < 0.01) with total and segmental fat mass as measured by bone densitometry [26]. This method provides an accurate evaluation of fat deposits across different body regions, including the abdomen, thigh, and upper arm, making it a reliable tool for body composition analysis in clinical and research settings [26]. This technique is especially helpful in single-patient clinical evaluations where a thorough analysis of fat distribution is needed [17].

The US is a valuable tool for detecting early indicators of inflammation within Hoffa’s fat pad. These include an increased thickness, with pathological values typically exceeding 10 mm in the sagittal plane, as well as changes in echotexture, such as hyperechogenicity compared with normal tissue and loss of the normal fibrillar pattern. An elevated Doppler signal is another key feature, with a resistive index (RI) below 0.6 indicating active neovascularization and inflammation. Additional findings include free fluid around Hoffa’s pad and the impingement sign, observed as compression of the fat pad during knee motion, which may appear before clinical symptoms manifest [22]. Early detection enables timely interventions that target inflammation, thereby preventing prolonged pain and reducing mobility restrictions [27]. Table 1 describes US changes in Hoffa’s fat pad according to different authors.

**Table 1:** Ultrasound findings of Hoffa’s fat pad in children with obesity

Author	Technique	Findings
Subhawong et al. [28]	B-mode ultrasound	- Fibrous areas: multiple foci of increased echogenicity. - Edema: homogeneous decrease in echogenicity.
Basha et al. [10]	Power Doppler	- Enhanced flow signals in the inflamed Hoffa’s fat pad, indicative of inflammation and neovascularization.
Satake et al. [33]	Elastography	- Fibrosis of Hoffa’s fat pad detected as increased elastographic signal.
Nalbant et al. [32]	Elastography	- Increased stiffness in thickened areas of the fat pad. - Softer signals in lesion areas. - Increased elasticity in edematous areas.
General Findings (Multiple Authors) [10, 28, 32, 33]	Combined techniques	- Presence of synovial fluid and synovial thickening associated with inflammation (Subhawong et al., Basha et al.). - Mechanical property changes in inflamed or edematous tissue areas, including stiffness variations (Nalbant et al., Satake et al.).
Satake et al. [33]	Combined techniques	- Highlights the primary role of ultrasound in managing inflammation in children with obesity, enabling targeted treatments

Longitudinal studies using magnetic resonance imaging (MRI) have demonstrated the association between Hoffa’s fat pad changes, such as reduced size, and the progression of knee osteoarthritis in patients with obesity. While MRI remains the gold standard for assessing detailed structural changes and early cartilage degeneration, US offers distinct advantages, particularly for real-time, dynamic evaluations and cost-effectiveness. Indeed, US is favored over MRI in clinical practice due to its greater accessibility, lower cost, and feasibility for real-time, dynamic assessments, making it an ideal first-line tool for the evaluation of joint inflammation in children with obesity. However, both modalities have unique strengths and limitations that influence their clinical application (Table 2) [23].

**Table 2:** Comparison of different imaging methods for Hoffa’s fat pad alterations

Aspect/Alteration	Ultrasounds	Magnetic Resonance	Elastosonography	Notes	Sources
Resolution	High resolution for superficial structures.	Superior resolution for deeper and complex soft tissues.	N/A	N/A	Albano et al., 2020 [23]
Dynamic Assessment	Allows real-time imaging during motion (e.g., impingement sign).	Limited to static imaging.	N/A	N/A	Albano et al., 2020 [23]
Cost	Low cost and widely available.	Expensive and less accessible.	N/A	N/A	Basha et al., 2020 [10]
Time Efficiency	Quick procedure (10–15 min).	Time-consuming (30–60 min).	N/A	N/A	Basha et al., 2020 [10]
Portability	Highly portable; can be used bedside.	Requires a fixed setup in specialized facilities.	N/A	N/A	Basha et al., 2020 [10]
Doppler Sensitivity	Identifies low-velocity blood flow (e.g., RI < 0.6).	Cannot evaluate blood flow directly.	N/A	N/A	Basha et al., 2020 [10]
Standardization	Operator dependent; variability in quality.	Consistent, standardized imaging.	N/A	N/A	Albano et al., 2020 [23]
Soft Tissue Assessment	Limited for deep structures.	Excellent for deeper structures like cartilage and ligaments.	N/A	N/A	Albano et al., 2020 [23]
Utility for Fat Pad	Effective for detecting inflammation (e.g., size > 10 mm, Doppler signal).	Ideal for detecting structural degeneration.	N/A	N/A	Vera-Perez et al., 2017 [22]
Thickening of Hoffa’s Fat Pad	Increased echogenicity and visible thickening.	Thickening and increased signal intensity in T2-weighted images.	Increased stiffness in elastography.	May indicate chronic inflammation or edema due to mechanical pressure.	Basha et al., 2020 [10]; Shin-Low et al., 2021 [30]; Manske et al., 2023 [34]; Shummalieva et al., 2023 [46]; Park et al., 2013 [51]; Subhawong et al., 2010 [28]
Lipid Infiltration	Mixed or elevated echogenicity areas	Increased signal intensity in T1-weighted sequences without fat suppression.	Heterogeneous elasticity with areas of softer consistency.	Obesity can lead to lipid accumulation, altering composition and mechanical properties.	Saxena et al., 2013 [9]; Park et al., 2013 [51]; Abelleyra et al., 2023 [54]
Fibrosis	Heterogeneous structure with areas of increased echogenicity.	Areas of low signal intensity in all sequences, indicating fibrotic tissue.	Increased stiffness in elastography, indicating fibrotic changes.	Develops due to chronic inflammation or repeated injuries, altering elasticity.	Shin-Low et al., 2021 [30]; Park et al., 2013 [51]; Gilliland et al., 2011 [52]; Calcaterra et al., 2020 [55]; Marginean et al., 2019 [56]
Lesions and Microlesions	Focal areas of altered echogenicity, potentially with signs of fiber discontinuity	Signal alteration areas with possible discontinuity in T2-weighted images.	Variable elasticity, possibly softer in lesion areas.	Acute or chronic lesions may be due to increased mechanical stress.	Saxena et al., 2013 [9]; Gilliland et al., 2011 [52]; Sakowicz et al., 2022 [57]; Manske et al., 2023 [34]
Edema	Homogeneous decrease in echogenicity.	Signal intensity increase in T2-weighted sequences, especially in fat-sat or STIR images.	Decreased stiffness or increased elasticity in edematous areas.	Indicates fluid accumulation and acute inflammation, altering elasticity.	Shin-Low et al., 2021 [30]; Basha et al., 2020 [10]; Gilliland et al., 2011 [52]; Sakowicz et al., 2022 [57]; Manske et al., 2023 [34]; Marginean et al., 2019 [56]
Altered Vascularization	Presence of increased flow signals on Doppler ultrasound.	Abnormal signal intensity or flow voids on contrast-enhanced MRI scans.	N/A	Indicates increased blood flow due to inflammation or neovascularization.	Basha et al., 2020 [10]; Herouvi et al., 2023 [53]
Area/Volume Alterations	Reduction in Hoffa’s fat pad size during progression of knee osteoarthritis.	Smaller size is associated with radiographic progression of knee osteoarthritis.	N/A	Alterations might include changes in overall volume, contour irregularities, or alterations in the fat pad configuration.	Calcaterra et al., 2020 [55]; Shummalieva et al., 2023 [46]; Testini et al., 2024 [58]

US imaging is a cornerstone method for describing the complex dynamics leading to joint inflammation. An increase in echogenicity in Hoffa’s fat pad and synovial membrane represents acute inflammatory cell accumulation, and changes in tissue composition are a direct sign of inflammation [10, 28]. Subhawong et al. described fibrous areas of Hoffa’s fat pad as an US feature of the heterogeneous structure containing multiple foci of increased echogenicity and areas of edema as a homogenous decrease in echogenicity [28]. The presence of synovial fluid and synovial tissue thickening is also related to inflammation status, which is characterized by swelling and joint pain [29]. Power Doppler improves the assessment of blood flow in the knee to detect areas of inflammation and neovascularization, indicating the body’s response to the metabolic needs of inflamed tissues [10, 30, 31]. Basha et al. reported an enhancement of inflamed Hoffa’s fat pad flow signals on Doppler US [10]. Nalbant et al. reported increased stiffness via elastography in the thickening of Hoffa’s fat pad, a softer signal in lesion areas, and decreased stiffness or increased elasticity in edematous areas [32]. Satake et al. identified Hoffa’s pad fibrosis as an increased elastography signal [33]. This development underlines the principal role of US in the assessment of knee health in children with obesity, thus deepening the knowledge of the disease and providing direction for specific therapeutic strategies [34].

Further advancements in US technology, including the integration of artificial intelligence (AI) and machine learning (ML), hold promise for addressing these limitations, potentially automating the detection and measurement of inflammatory changes, improving diagnostic accuracy, and reducing operator-dependent biases. AI and ML may also narrow the gap between US and MRI in terms of reliability for the early detection of subtle structural changes, monitoring disease progression [35–37], and potentially providing new possibilities for improving the health outcomes of children with obesity-related musculoskeletal disorders [38, 39].

## Clinical applications in the management of pediatric knee inflammation

US is both a diagnostic tool, detecting inflammatory changes before the onset of clinical symptoms, and a therapeutic approach for pediatric patients with obesity-induced knee inflammation [24, 40]. This proactive approach can support healthcare providers in the initiation of timely treatment interventions, possibly by arresting the inflammatory process and avoiding more severe outcomes. Prognostically, US findings such as Doppler signal intensity and grayscale changes in the fat pad and joint structures can guide treatment adjustments and monitor therapeutic responses. Pratt et al. [41] highlighted that the integration of US in early arthritis clinics improved the accuracy of persistent inflammatory arthritis predictions in pediatric patients. Furthermore, US elastography can detect early changes in fat pad stiffness; increased in the case of fibrotic tissue or decreased in edematous areas, correlating with specific pathological states, aiding in targeted interventions [32]. Studies have demonstrated that US-detected synovial changes, such as increased Doppler signals and synovial hypertrophy, are strongly correlated with the severity of knee osteoarthritis and associated pain. Synovial hypertrophy has been identified as an independent predictor of knee pain severity and functional impairment; Wu et al. [42] demonstrated that participants presenting this sign showed a risk of experiencing pain six times greater. In addition, from a prognostic perspective, the presence of synovial inflammation visualized by US is associated with poorer long-term outcomes in patients with knee osteoarthritis, including a greater risk of persistent inflammation, pain, radiographic progression, and reduced therapeutic efficacy over time [43]. Power Doppler US has shown a significant ability to assess synovial perfusion changes after intra-articular corticosteroid injections, with notable reductions in Doppler signals corresponding to reduced inflammation and improved clinical outcomes [44].

**Fig. 2:**
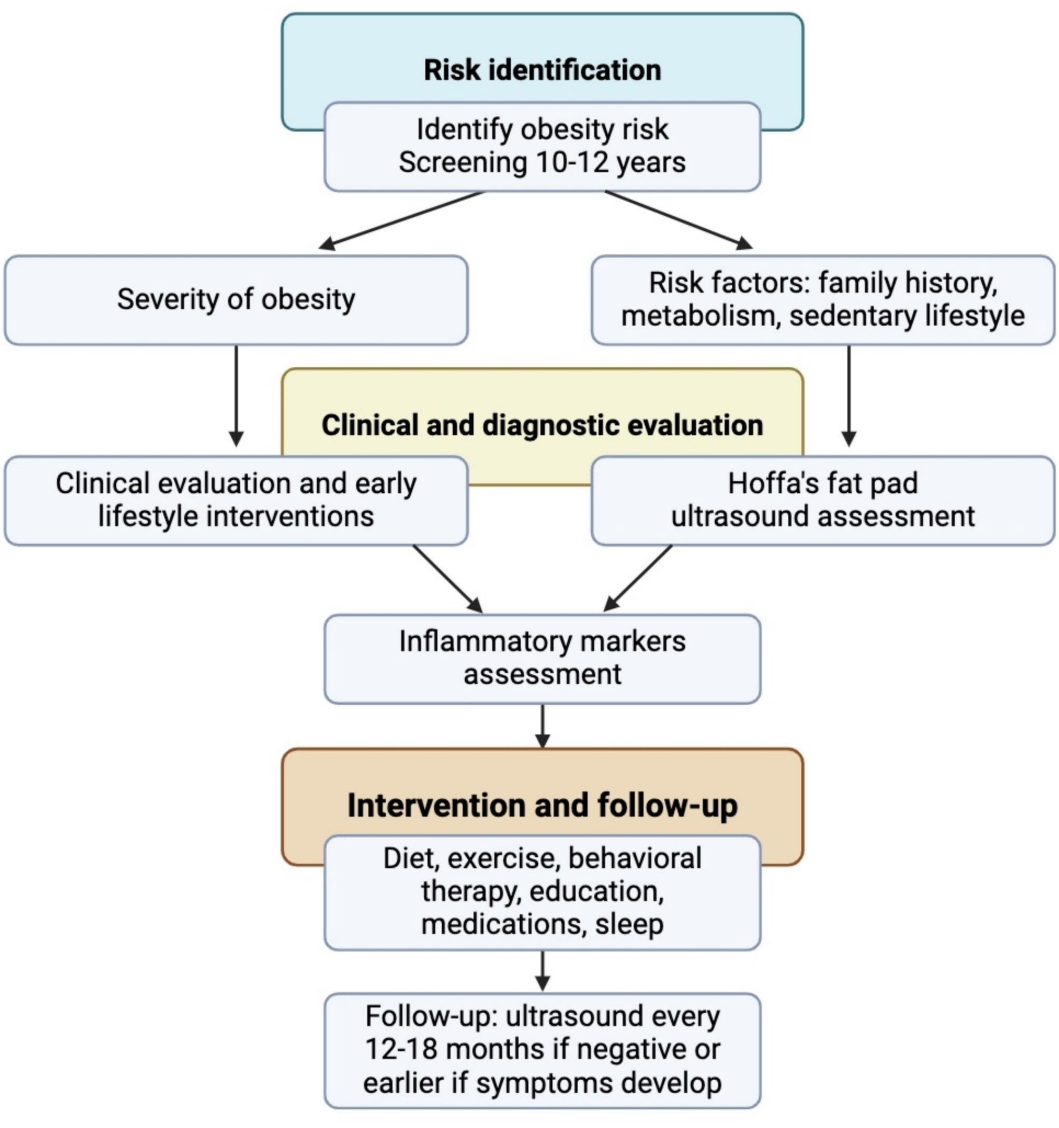
Comprehensive management approach for pediatric obesity and joint inflammation

In addition to monitoring, US imaging changes the accuracy of treatment administration. Its application in guiding intra-articular injections appears to be a significant advancement [45], enabling the precision and safety of the administration of drugs directly to inflammation sites [30]. Visual guidance facilitates accurate needle positioning, which enables therapeutic agents to be delivered directly to the target site, thereby optimizing the treatment outcome and improving the recovery outlook [31]. This approach aimed at precision not only improves the results of treatment but also decreases the risk of side effects, making it an essential tool in the management of obesity-associated knee inflammation [31]. In addition to obesity, factors such as family history, sedentary lifestyle, and metabolic abnormalities may influence joint inflammation risk, while the management approach may vary according to the degree of obesity. US screening for joint inflammation in children with obesity may be most beneficial when initiated at the onset of puberty (approximately 10–12 years of age), as hormonal changes during this period can amplify inflammatory responses. Early identification through such targeted screening could allow for timely interventions and help mitigate progressive joint damage. In cases where initial US findings are normal, reassessment could be considered every 12–18 months or sooner if clinical symptoms develop. Figure 2 represents a possible flowchart for the management of pediatric obesity-related joint inflammation.

## Significance of early intervention

US-guided interventions usually concentrate on weight loss methods to alleviate joint overload and lower the systemic inflammation associated with obesity [46]. Dietary modifications, physical exercise, and behavior therapy have been found to be successful in increasing weight reduction and monitoring US markers of Hoffa’s fat pad inflammation [46].

Concomitantly, anti-inflammatory pharmacological approaches have been considered in combination with lifestyle changes. The management of symptomatic knee conditions in obesity has been marked by the administration of nonsteroidal anti-inflammatory drugs (NSAIDs) [46]. Zeng et al. [47] reported that NSAIDs may change the US features of Hoffa’s fat pad by reducing inflammation and blood flow in the area. Moreover, physical therapy is usually advised for the implementation of rehabilitation exercises targeted at strengthening the muscles surrounding the knee joint [48]. These treatments are aimed at enhancing joint function and reducing the impact of obesity on Hoffa’s fat pad, as revealed by positive alterations in US imaging parameters [48]. A delay in identifying inflammation in Hoffa’s pad may trigger a series of structural changes in the knee joint, leading to some complications [49].

US-guided intra-articular injections represent a major improvement in the precision and safety of therapeutic interventions. By providing real-time visualization of anatomical landmarks, US enables clinicians to accurately target inflamed tissues, minimizing complications and maximizing therapeutic efficacy [45].

The benefits of US guidance are evident in the administration of corticosteroids and hyaluronic acid injections, which minimize procedural discomfort and reduce the psychological burden often associated with interventions in children [50]. It has been proven that US guidance reduces the risk of misplacement, achieving over 95% accuracy in delivering medication to the intra-articular space compared with blind techniques, which have a success rate as low as 70% in patients with obesity [51].

**Fig. 3:**
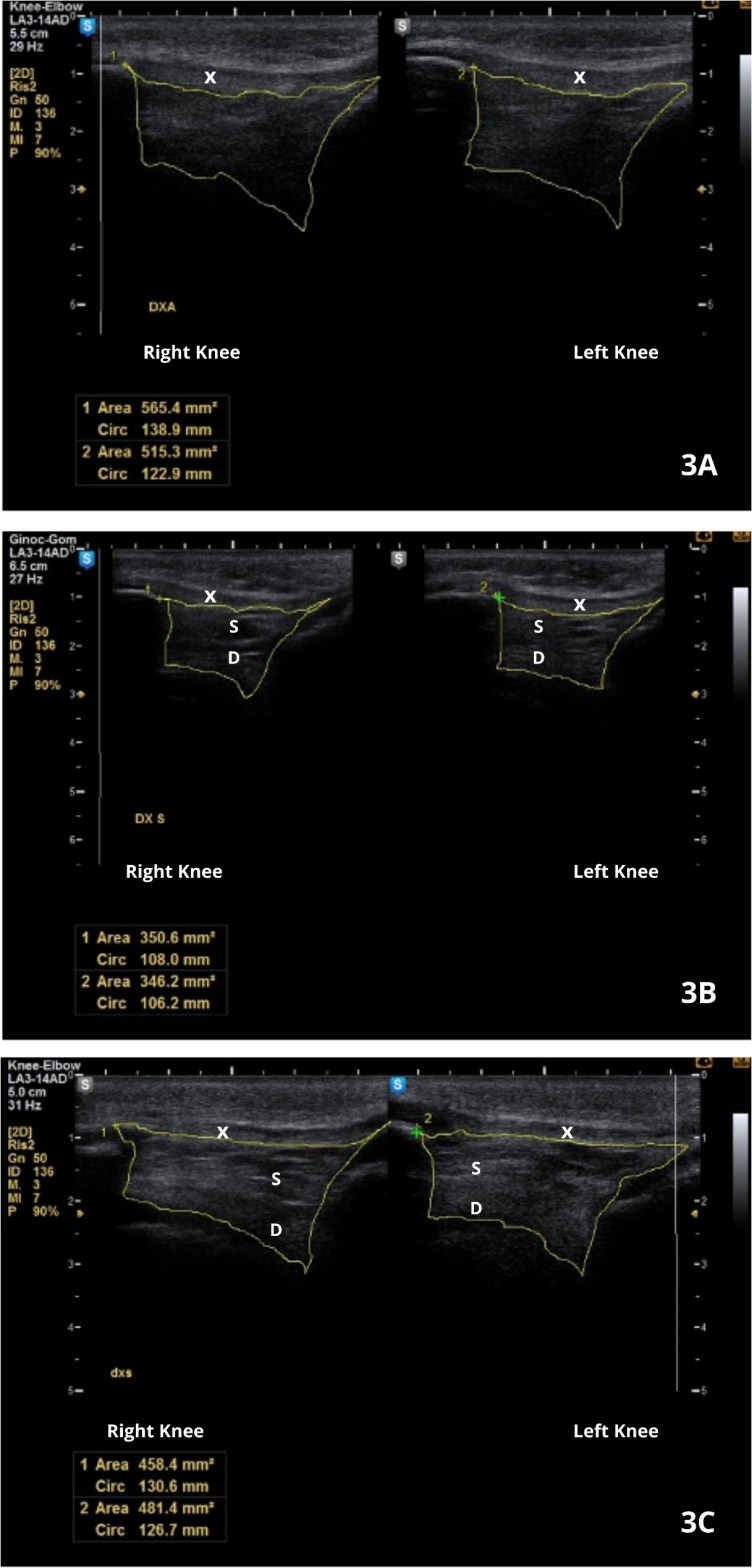
US evaluation of Hoffa’s fat pad. **3 A-3 C** B-mode US images of the knee on the longitudinal subpatellar scan plane, which provide an evaluation of Hoffa’s fat pad (area outlined in yellow) echostructure and echogenicity, compared to the patellar tendon (X). All patients were 9-year-old prepubescent females. **3 A** Patient with higher BMI (BMI = 29.31, > 99th percentile) presents with hypoechoic fat pads with unidentifiable double-layer structures and larger fat pad areas (565.4 mm² right, 515.3 mm² left), potentially indicating increased tissue volume and altered composition due to greater mechanical or inflammatory stress. **3B**,**3 C** Patients with lower BMIs (respectively BMI = 23.13, 95–99th percentile, **3B**; BMI = 21.41, 85–95th percentile, **3 C**) presented slightly hypoechoic fat pads with identifiable superficial septate (S) and deep homogeneously hypoechoic (D) layer structures and smaller fat pad areas (350.6 mm² right, 346.2 mm² left for BMI 23.13), which may reflect reduced mechanical stress and more preserved tissue characteristics. The OMERACT US semiquantitative scoring system revealed no signs of inflammation, such as effusion or hypervascularization, in any of these patients. Abbreviations: BMI, body mass index; OMERACT, Outcome Measures in Rheumatology; US, ultrasound

US-guided procedures also facilitate minimally invasive interventions, such as aspiration of joint effusion, drainage of Baker’s cysts, and biopsies of synovial tissue. These procedures are critical in both diagnostic confirmation and therapeutic management and may reduce the need for more invasive surgical approaches. By ensuring precise drug delivery and avoiding damage to adjacent structures, US-guided procedures optimize treatment outcomes while maintaining patient safety and comfort [52]. Lifestyle changes and pharmacological interventions are crucial for preventing long-term musculoskeletal complications in children by addressing underlying obesity and its inflammatory effects [53]. Figure 3 provides examples of an US evaluation of infrapatellar Hoffa’s fat pads.

## Conclusions

Obesity is not just a deposit of extra fat but also an active endocrine tissue that releases different adipokines and triggers inflammatory responses, potentially leading to complications such as synovitis, cartilage degeneration, joint soft tissue damage, and osteoarthritis. Indeed, it is not merely the excess fat, but rather the active endocrine function of adipose tissue, through the secretion of proinflammatory cytokines, that plays a pivotal role in joint inflammation. The infrapatellar fat pad is highly sensitive to the adverse effects of obesity, with the infiltration of immune cells producing proinflammatory cytokines playing a key role in the pathogenesis of joint disease and related complications. Hypertrophy with reduced echogenicity is the typical US finding of acute inflammation and may serve as a predictive marker for the development of joint destruction. The overload of mechanical pressure on joints leads to microtrauma, intensifying the inflammatory reaction and worsening joint degeneration. Moreover, these changes seem to be reversible, as shown by weight loss interventions that decrease US signals.

US is a noninvasive, accessible method for detecting and monitoring Hoffa’s fat pad inflammation. Based on the current literature, while US screening for knee joint inflammation in children with obesity shows promise, the evidence is not yet sufficient to recommend routine screening for all joint regions. Further studies are needed to determine the optimal screening protocols and to establish whether a targeted approach. The associations among US findings, obesity, and inflammation therefore emphasize the necessity of future research on specific interventions and possible changes in clinical practices to reduce the effect of the epidemic of obesity on children’s joint health.

## Data Availability

Not applicable.

## Abbreviations

BAI: Body Adiposity Index
BMI: Body Mass Index
FMI: Fat Mass Index
IL-6: Interleukin-6
MRI: Magnetic Resonance Imaging
NSAIDs: Nonsteroidal Anti-inflammatory Drugs
RI: Resistive Index
TNF-α: Tumor Necrosis Factor-alpha
US: Ultrasound
